# A dataset for monitoring agricultural drought in Europe

**DOI:** 10.1038/s41597-024-04199-8

**Published:** 2025-02-20

**Authors:** Guido Fioravanti, Andrea Toreti, Carmelo Cammalleri, Carolina Arias Muñoz, Davide Bavera, Alfred De Jager, Arthur Hrast Essenfelder, Chiara Di Ciollo, Dario Masante, Diego Magni, Juan Acosta Navarro, Marco Mazzesch, Willem Maetens

**Affiliations:** 1 European Commission, Joint Research Centre. Via Enrico Fermi, 2749. 21027, Ispra, VA Italy; 2 Istituto Superiore per la Protezione e la Ricerca Ambientale, Rome, Italy; 3 Politecnico Milano, Dipartimento di Ingegneria Civile e Ambientale (DICA), Milano, 20133 Italy; 4 Arcadia SIT, Milano, Italy; 5 Unisystems, Milano, Italy; 6 Unisystems, Luxembourg Sàrl, Bertrange, Luxembourg

**Keywords:** Natural hazards, Hydrology

## Abstract

This paper introduces the Combined Drought Indicator dataset, a collection of raster maps generated by the Copernicus European Drought Observatory for monitoring agricultural drought in Europe. Computationally, the CDI involves three indicators: the Standardized Precipitation Index, the Soil Moisture Anomaly and the Fraction of the Photosyntetically Active Radiation anomaly. These are complemented by the use of crop and snow masks. The CDI dataset has a spatial resolution of 1/24 decimal degrees (∼5 km), a temporal resolution of 10 days and is available from 2012 onward. As drought effects are variegated both in space and time, the CDI provides an effective instrument for assessing the different stages of propagation of agricultural drought and their spatial extent. Furthermore, the CDI maps provide relevant information for those private and public actors (water resource agencies, farmers, land managers and so on) involved both in drought preparedness and planning to mitigate drought impacts. Users can access and download the dataset from the Copernicus European Drought Observatory web portal, where an online mapviewer and clickable maps facilitate its interactive exploration.

## Background & Summary

In the last decades, drought events have been worldwide increasing in frequency and duration, as well as their impacts on human and natural systems, with more than 2.3 billion people facing with water stress^1^. The year 2022 has been by far an exceptional one for drought: long periods of water shortage and record-high temperatures have dominated large areas of the globe, particularly in Europe^2,3^. As evidence of this, the Copernicus Emergency Management Service’s Global Drought Observatory (GDO, https://drought.emergency.copernicus.eu/) published a number of analytical reports to document the severe dry conditions across the western Mediterranean region in the first two months of 2022, the broad and persistent drought affecting the coastal regions of Europe and East Africa, the severe hydrological drought over the Yangtze River basin in China in August-September 2022 and the dry meteorological conditions at the end of September 2022 across the Parana-La Plata Basin in Brasil-Argentina.

Drought is commonly described as a “complex” natural phenomenon^4^. First, drought events can occur almost anywhere, not only in regions perceived by many as dry, but also in areas characterized by a surplus of water^4,5^. Second, while drought conditions usually emerge gradually over time and persist for long periods^6^, rapid events (“flash droughts”) are also possible^7^. As a result, the social and environmental effects of drought can vary considerably in space and time^8,9^. Finally, due to climate change, the frequency and severity of drought events show a tendency to increase that will affect many parts of the world over the 21st century^10,11^.

Drought is usually identified with a period characterized by unusually persistent dry weather conditions affecting the hydrological balance. Due to its inherent complexity, different phases/types can be identified and described by using a set of variegated hydro-meteorological and biophysical parameters. It is common to distinguish at least three phases of drought^12^: “meteorological drought” (a period characterized by a prolonged deficit of precipitation), “agricultural drought” (when declining soil moisture conditions affect crops and vegetation) and “hydrological drought” (when the deficit of water affects rivers, lakes, reservoirs and groundwater).

Monitoring agricultural drought is crucial as drought events can significantly impact agricultural production and food systems^13^. At the same time, this is a non-trivial task given the uncertainty of the drivers (precipitation, temperature, etc.) and considering that the complex propagation from meteorological to agricultural and, eventually, to hydrological drought is also influenced by local climate and catchment characteristics^14^. For this reason, drought early warning systems usually relies on the integration of multiple sources of information to complement precipitation data^15,16^. For example, Enenkel *et al*.^17^ describe an agricultural drought indicator for Ethiopia (the Enhanced Combined Drought Index) based on satellite-derived observations of rainfall, soil moisture, land surface temperature and vegetation vigor. Likewise, the VegDri index is a drought indicator for the conterminous United States, which integrates remote sensing, climate, and biophysical data^18^. Finally, the Vegetation-Soil Water Deficit method in an indicator introduced by Cao *et al*.^19^ to better assess agricultural drought conditions in Northeast China using precipitation, potential evapotranspiration and soil moisture.

In order to monitor and capture different aspects of drought conditions in Europe, the Copernicus European Drought Observatory (EDO, https://drought.emergency.copernicus.eu/) managed by the European Commission’s Joint Research Centre (JRC) provides and timely updates a wide range of spatial indicators, including an indicator for agricultural drought: the Combined Drought Indicator (CDI). The CDI is used to identify areas suffering from various degrees of drought (areas at risk of agricultural drought, areas where the vegetation has already been affected and areas recovering from drought) and their spatial extent. As the name suggests, the CDI integrates three complementary scientific indicators: the Standardized Precipitation Index (SPI), the Soil Moisture Anomaly (SMA) and the Fraction of the Photosyntetically Active Radiation (FAPAR) Anomaly.

The Combined Drought Indicator dataset is a collection of raster maps regularly updated every 10 days (10-day). This is a reasonable frequency for monitoring agricultural drought, given that only 10–20 days of water stress could severely affect the final crop production^20,21^. Table 1 summarizes the main characteristics of the CDI.

**Table 1:** Main characteristics of the Combined Drought Indicator.

Indicator	Long Name	Temporal Coverage (Resolution)	Spatial Coverage (Resolution)	Format
CDI	Combined Drought Indicator	2012 - today (10-day)	Europe (1/24 decimal degrees, ∼5 km at the equator)	netCDF/GeoTiff

Since 2012, the CDI has been a cornerstone of the EDO and GDO’s drought analytical reports, providing essential insights and severity assessments of drought conditions. The CDI importance is further evident in the public engagement: according to the EDO and GDO website internal analytics, the EDO portal recorded over 20.000 visits in 2023, demonstrating the dataset’s value to the community.

The CDI was initially developed by Sepulcre-Canto *et al*.^22^, while a first major revision (usually referred as the CDI version 2, CDIv2 hereafter) was proposed by Cammalleri *et al*.^23^. Since 2021, the CDI maps and data have been produced operationally and are freely available to final users through the EDO web portal. In March 2023, the version 3 of the CDI (CDIv3, hereafter) was released. This update mainly benefits from the incorporation of static crop masks, which prevent the assessment of vegetation stress in crop areas out of the growing season (namely, areas where the crops have not been sown or where they are dormant). The release of the version 4 (CDIv4, December 2023) took a further step forward in advancing the performance of the Combined Drought Indicator, with the integration of a collection of dynamic (time-varying) snow masks for resolving unrealistic model-based negative soil moisture anomalies in presence of snow cover.

The use of spatial data to assess drought conditions enables a large-scale and timely monitoring system which conveys scientific expertise to mainstream media and final users involved both in drought preparedness and planning to mitigate drought impacts (water resource agencies, farmers, land managers, city councils and so on). Through the modern and user-friendly interface of our data portal, users can dynamically explore the CDI maps and compare them in space and time. In addition, the downloadable data series enables users to run further analysis, generate written reports, or create ad-hoc maps and graphics. The relevance of the CDI dataset for drought monitoring and early warning provides the motivation for this paper. Here we review its main characteristics.

## Methods

The CDI assumes that the development of agricultural drought is a top-down (hierarchical) linear process, where a shortage of precipitation (Watch stage) triggers declining soil moisture conditions (Warning stage) which, in turn, promotes negative effects on vegetation (Alert stage). Nonetheless, as explained later in the text, the CDI is also able to partly manage non-linearities in the drought process development (e.g. in the column C or in the recovery classes in the column A of the CDI conceptual framework illustrated in the following section).

The Combined Drought Indicator adopts a qualitative scale of seven classes to track and compare the different stages of agricultural drought spatially (Table 2). From a computational point view, each pixel in a CDI map summarizes: 1) the simultaneous values of three anomaly indicators (SPI, SMA and FAPAR) at the 10-day period **T**; 2) the CDI class assigned to the same pixel in the previous 10-day period **T-1**. Using these two pieces of information, the CDI algorithm classifies each pixel in the study domain according to the seven classes of Table 2.

**Table 2:** The Combined Drought Indicator classes and their interpretation. .

**Fig. 1:**
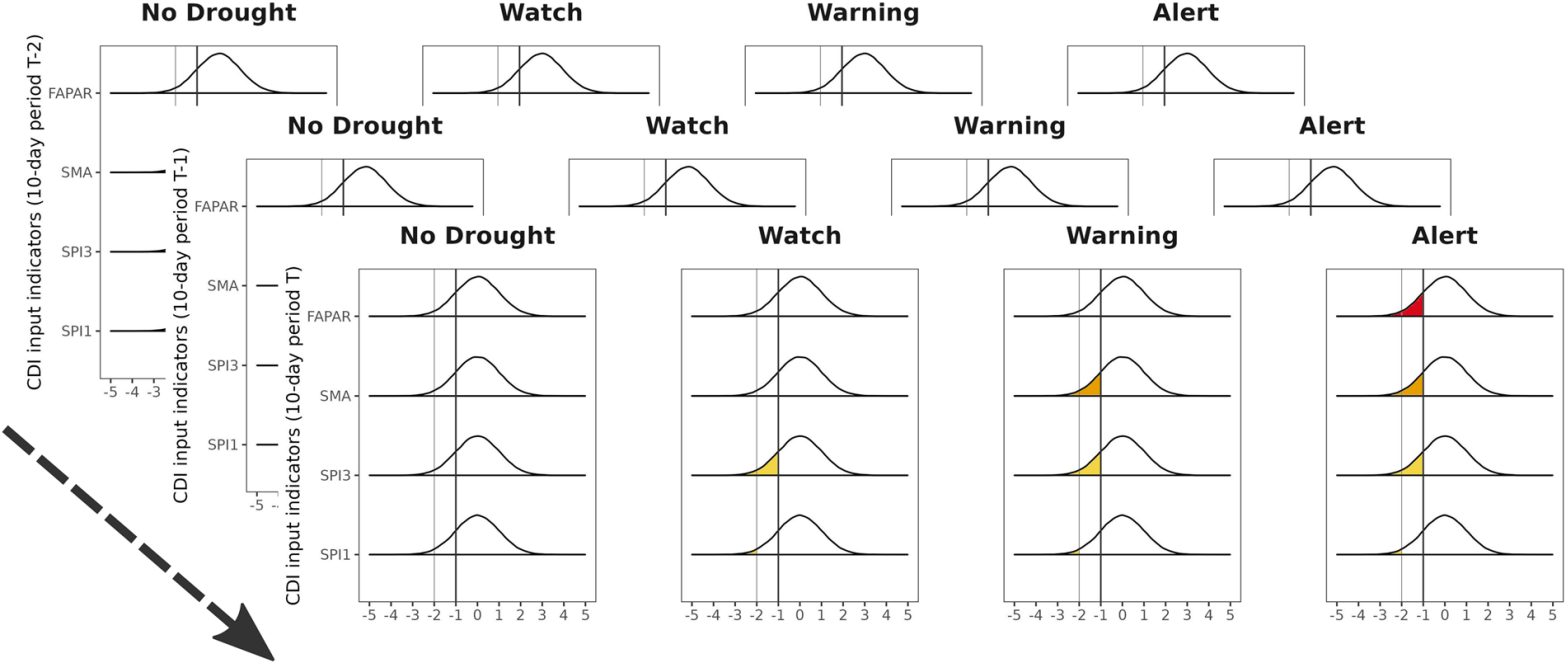
The computation of the CDI involves three input indicators: SPI, SMA and FAPAR anomaly. From a mathematical point of view, the three input indicators are represented in terms of symmetric, bell-shaped distributions with zero mean and unit standard deviation. The CDI monitors if one or more indicators (at the 10-day period **T**) are below their corresponding reference thresholds. This information is then integrated with the CDI class at the 10-day period **T-1** in order to classify each pixel of the study domain according to a seven-category qualitative scale. Note, the diagram shows the SPI calculated on two distinct time scales: ∼30 days (SPI-1) and ∼90 days (SPI-3).

**Fig. 2:**
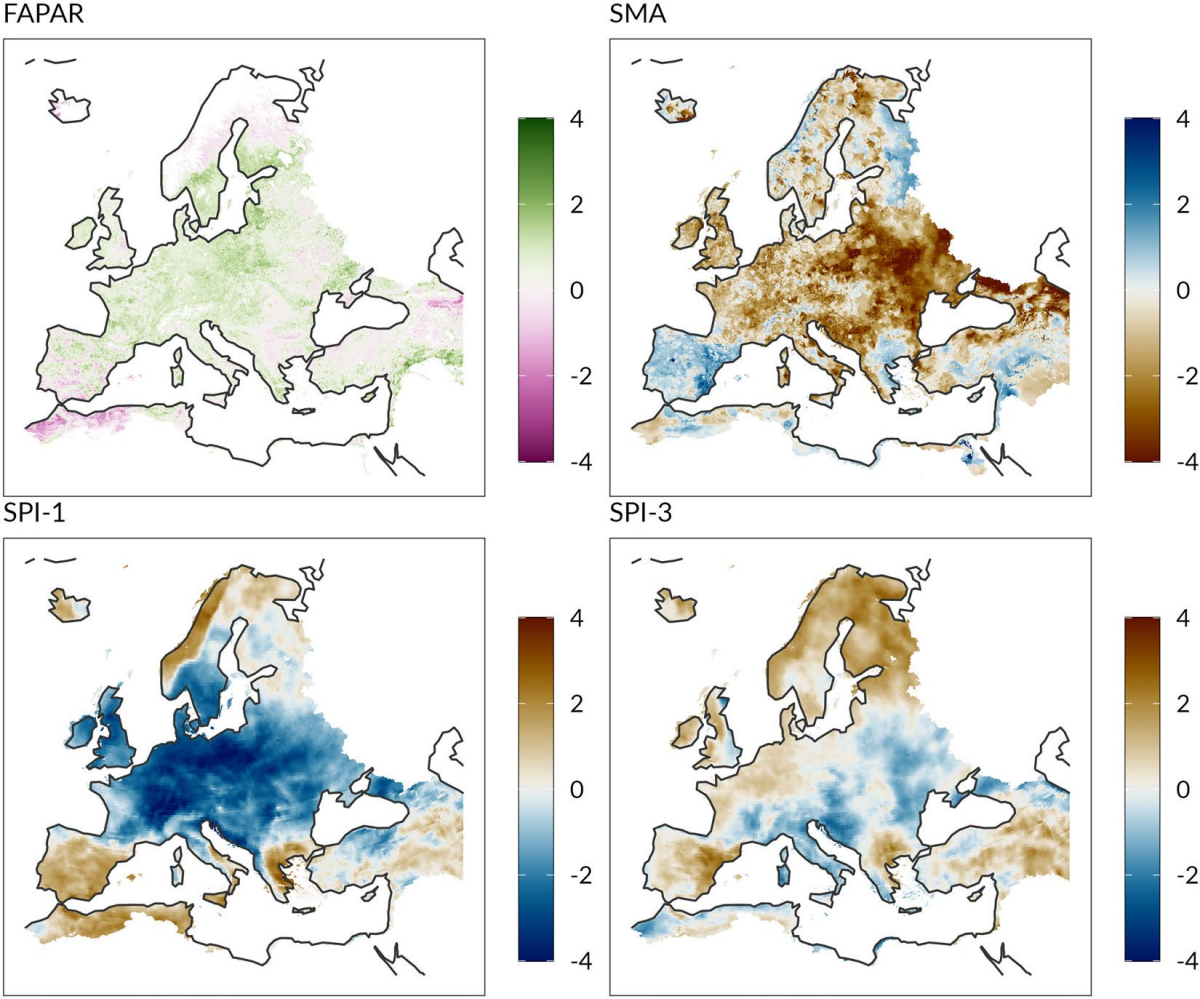
An example of input indicators (FAPAR anomaly, SMA, SPI-1 and SPI-3) used in computing the Combined Drought Indicator for the second 10-day period of April. Note, the four indicators have dimensionless units.

Precipitation, soil moisture and vegetation are the three main components which contribute to the definition of the CDI (point 1 in the above-mentioned list). These enter into the computation of the CDI in form of anomalies. The reason for using anomalies is to put “*what we observe today*” in a reference context. Indeed, the purpose of the SPI, SMA and FAPAR is to monitor and quantify deviations (anomalies) from normal long-term conditions (mean) in total precipitation, the degree of soil moisture in the soil and the vegetation health status. Each time one of the above-mentioned indicators falls below (exceeds) its corresponding critical threshold, the CDI signals that drought conditions have changed from normal (no drought) to one of the three primary drought classes: Watch, Warning and Alert. A visual representation of the three input anomaly indicators is provided in Figs. 1 and 2.

One major feature of the CDI is the presence of three recovery classes: Recovery, Temporary Soil Moisture Recovery and Temporary Vegetation Recovery. These represent a temporary stage of transition from drought (Watch, Warning, Alert) to “no drought”. The purpose of the recovery classes is twofold. First, they describe “after drought” conditions, namely, pixels where FAPAR anomalies and/or SMA are still negative (after a drought event) but above the reference threshold; second, they allow to partly account for potential asynchronies between spatial anomalies in precipitation, soil moisture and vegetation^23^. This point is illustrated through an example in the next section.

### General CDI conceptual framework

The conceptual framework of the CDI is a decision table (Table 3) which visualizes all the possible combinations among inputs at time step **T-1** and **T**, as well as their related outputs. Indeed, in a CDI map each pixel must be intended as the result of an algorithm which implements the logic in Table 3.

**Table 3:** CDI conceptual framework. .

Note, to improve the readability of the table, we use “0” and “1” as concise representations of “(SPI-1 > -2) AND (SPI-3 > -1)” and “(SPI-1 < -2) OR (SPI-3 < −1)”.

The CDI conceptual framework is divided into eight-lettered columns (A through H) and seven-numbered rows. Each column details how the three input anomaly indicators SPI, SMA and FAPAR intersect at time step **T**; the rows indicate the drought conditions (CDI levels) at time step **T-1**; the cells (the combination among columns and rows) return the CDI level at time step **T**. In each column the variables are arranged in a hierarchical structure which reflects the propagation process from meteorological drought to agricultural drought (SPI $\Rightarrow$ SMA $\Rightarrow$ FAPAR). In addition, for each indicator the reference thresholds are reported in the column headings.

Here, we provide some examples to clarify how the CDI conceptual framework works. The columns G and H describe pixels that, at time step **T**, are characterized by abnormal (below the reference threshold) anomalies both in the precipitation level (SPI) and the vegetation health status (FAPAR anomaly). Likewise, column E corresponds to pixels with abnormal anomalies both in the degree of soil moisture (SMA) and the vegetation health status. Whatever the value of these pixels at time **T-1**, the CDI assigns Alert as the output class at the time step **T**. The logic is that the columns E, G and H describe conditions where dryness has severely affected the vegetation health status. In contrast, the column A describes pixels where the three spatial indicators are simultaneously above their reference thresholds at time **T** and the CDI outcome is dependent on the values of the same pixels at time **T-1**. Interestingly, the column A clearly shows that the CDI logic does not allow a direct transition from drought (Watch, Warning and Alert) to “No drought” conditions. Such transition is always modulated through three recovery classes. A pixel classified as “Temporary Soil Moisture Recovery” is a pixel with: (1) abnormal soil moisture anomalies at time **T-1**; (2) soil moisture anomalies in the range $(-\mathrm{0.5;0]}$ at time **T**. This means that, at time **T**, the soil moisture anomalies are still negative but closer to 0 (no drought) than to the reference threshold of -1. The same interpretation is valid for the “Temporary Vegetation Recovery” class.

We conclude this section discussing the column C. This corresponds to pixels with negative ($\le -1$) soil moisture anomalies belonging to the drought class “Warning”. The interesting point here is the logical condition ((SPI-1 > 0.5) AND (SPI-3 > 0)) and its negation (!(SPI-1 > 0.5) AND (SPI-3 > 0)). These define two corresponding sub-classes for the SPI. The first one describes wet precipitation conditions which counterbalance the negative soil moisture anomalies when the CDI at time **T-1** is “No Drought” or “Recovery” (in this case, the CDI output class is “No Drought”). The second one describes conditions where the precipitation levels are not high enough to counterbalance the negative soil moisture anomalies (in this case, the CDI output class is “Warning”).

To summarize, the CDI assumes that agricultural drought originates from persistent lack of precipitation, affects negatively the soil moisture and, ultimately, results in reduced photosynthetic activity (vegetation stress) and crop failure. Nonetheless, the CDI implementation is able to manage also more complex situations characterized by nonlinear relationships among SPI, SMA and FAPAR.

### CDI input indicators

The computation of the Combined Drought Indicator relies on different data sources (Tables 5, 6 and 7), which in turn involve different spatial and temporal resolutions, geographic projections and data formats. Dealing with datasets of varying horizontal resolutions and time spans presents several challenges (e.g. the potential issues due to interpolation errors). Therefore, careful consideration of appropriate methods for regridding and aggregating the data consistently has been a priority. The main details of the data processing are provided in the following sections.

**Table 4:** The table shows the days in a month associated with each time step (10-day period) **T**.

Time steps in a month	Days	Month
First time step	1–10	All months
Second time step	11–20	All months
	21–31	January, March, May, July, August, October, December
Third time step	21–30	April, June, September, November
	21–28/29	February

**Table 5:** Main characteristics of the Standardized Precipitation Index.

Indicator	Long Name	Baseline	Threshold	Mask
SPI-1	Standardized Precipitation Index at 30 days	1981–2010	−2	—
SPI-3	Standardized Precipitation Index at 90 days		−1	—
**Input data for SPI**	**Data source**	**Temporal Coverage (Resolution)**	**Spatial Coverage (Resolution)**	**Format**
	ERA5 reanalysis	1940 - today (Hourly)	Global (31 km)	netCDF

**Table 6:** Main characteristics of the Soil Moisture Anomaly.

Indicator	Long Name	Baseline	Threshold	Mask
SMA	Soil Moisture Anomaly	1995 - onward	−1	Snow Masks
**Input data for SMA**	**Data source**	**Temporal Coverage (Resolution)**	**Spatial Coverage (Resolution)**	**Format**
	LISFLOOD model	1995 - today (Daily)	Europe (0.016 decimal degrees, ∼5 km st the equator)	netCDF

**Table 7:** Main characteristics of the Fraction of the Absorbed Photosyntetically Active Radiation anomaly.

Indicator	Long Name	Baseline	Threshold	Mask
FAPAR	Fraction of the Absorbed Photosyntetically Active Radiation anomaly	2012 - onward	−1	Crop Masks
**Input data for FAPAR anomaly**	**Data source**	**Temporal Coverage (Resolution)**	**Spatial Coverage (Resolution)**	**Format**
	VIIRS	2012 - today (8-day)	Global (500 m)	HDF

### Standardization of the input indicators

For the development of the CDI, precipitation, soil moisture and vegetation data records are standardized to have zero mean and unit variance.

For soil moisture and vegetation, the standardization in based on the following formula:

1 $${Z}_{T,Y}^{i}=\frac{{I}_{T,Y}^{i}-{\mu }_{T,Baseline}^{<mml:mpadded xmlns:xlink="http://www.w3.org/1999/xlink" lspace="-2.5pt">i</mml:mpadded>}}{{\sigma }_{T,Baseline}^{i}}$$

where ${I}_{T,Y}^{i}$ is the value of the non-standardized indicator at location (pixel) $i=\mathrm{l,}\ldots ,np$, time $T=\mathrm{l,}\ldots ,36$ and year $Y=\mathrm{2012,}\ldots ,Today$. As detailed below, the generic quantity ${I}_{T,Y}^{i}$ can correspond to:

- the 10-day average of the Soil Moisture Index (for the Soil Moisture Anomaly computation);
- the 10-day composite of the VIIRS FAPAR products (for the FAPAR anomaly computation).

The number of time steps **T** in a year $Y$ is 36, three per month. Considering that each time step spans approximately 10 days (Table 4), we generically refer to **T** as a “10-day” period.

$np$ is the number of pixels in the CDI study domain (a number of around 363000 units covering a surface of approximately 900000 ${{\rm{km}}}^{2}$). ${\mu }_{T,Baseline}^{<mml:mpadded xmlns:xlink="http://www.w3.org/1999/xlink" lspace="-2pt">i</mml:mpadded>}$ and ${\sigma }_{T,Baseline}^{i}$ are the average and standard deviation, respectively, for the location $i$ and time step **T**. These are calculated over a reference period ($Baseline$) which depends on the indicator of interest ${I}_{T,Y}^{i}$. Finally, ${Z}_{T,Y}^{i}$ is the z-score, the standardized indicator (SMA, FAPAR) expressed in units of standard deviations (anomalies) from the long-term mean ${\mu }_{T,Baseline}^{<mml:mpadded xmlns:xlink="http://www.w3.org/1999/xlink" lspace="-2pt">i</mml:mpadded>}$.

As for precipitation, the standardization follows a different approach (see next subsection). Nonetheless, ${I}_{T,Y}^{i}$ must be intended as the cumulated precipitation over a given timescale which terminates in **T** and ${Z}_{T,Y}^{i}$ (the SPI) as the cumulated precipitation standardized to a normal distribution having 0 mean and unit standard deviation.

Standardization facilitates comparability across space and time, as well as among the three indicators themselves. In addition, standardization allows to interpret each indicator as follows: a value close to 0 indicates that the indicator resembles “normal”, long-term conditions; a value close to or below -1 indicates an abnormal departure from average, as a result of worsening drought conditions.

### Standardized precipitation index

Agricultural drought usually is the result of a prolonged precipitation deficit (meteorological drought)^24^. The Standardized Precipitation Index (SPI) is a well-know metric recommended by the World Meteorological Organization (WMO) for meteorological drought monitoring^25^. Generally speaking, the SPI quantifies the deviation of the cumulated precipitation on a given timescale (here, ∼30 and ∼90 days) with respect to a reference baseline. However, as precipitation data are usually characterized by asymmetrical skewed-to-the-right frequency distributions, the computation of standardized anomalies cannot be based on Equation 1. For the SPI computation, the first step is to fit the cumulated precipitation data to an appropriate probability distribution function (here, the gamma distribution) and then transform it into a normal cumulative density function. A detailed description of the SPI is available in^26^.

There are two points in this work that deserve attention. First, the SPI computation is based on a 30-year reference period (1981–2010), instead of using an “as much data as possible” reference period. The use of 30-year periods for the SPI computation has been advocated by several recent studies^27^. Second, the CDI monitors precipitation deficit at two different short timescales: a 30-day (SPI-1) and a 90-day timescale (SPI-3). The rationale for their use is that soil moisture conditions respond fast to dryness^28,29^. The CDI assesses below-normal conditions in the precipitation level when one or both of these two indicators fall below their corresponding critical thresholds: a value of -1 for the SPI-3 (“Moderate meteorological drought”) and -2 for the SPI-1 (“Extreme meteorological drought”)^30^.

The SPI-1 and SPI-3 maps (Table 5) are calculated using precipitation data from the ERA5 (European ReAnalysis 5th Generation) reanalysis^31^. ERA5 is a dataset published by the European Centre for Medium-Range Weather Forecasts (ECMWF) which provides hourly estimates of a large number of atmospheric, land and oceanic climate variables from 1940 onwards. ERA5 data have been evaluated and applied in different regions of the world^32–34^. As for the European domain, ERA5 precipitation estimates show good agreement with observations even for extreme precipitation^35^. ERA5 precipitation reanalysis are archived on a reduced Gaussian grid (linear grid) with a resolution of around 31 km. The netCDF archives are available in the Copernicus Climate Data Store (https://cds.climate.copernicus.eu) on regular latitude-longitude grids at 0.25° × 0.25° resolution already referenced in the horizontal with respect to the WGS84 ellipse (EPSG:4326 geographic coordinates). For the temporal aggregation of the original hourly data to the monthly time scale, we used the Climate Data Operator command line tool^36^.

### Soil moisture anomalies

Soil moisture is one of the most relevant component for agricultural drought monitoring because growth conditions of crops and vegetation mainly depend on the water stored in the soil^37^. Soil moisture information is usually provided by: (1) *in-situ* measurements; (2) land/hydrological models, and (3) satellites. The SMA component of the CDI (Table 6) is calculated using model-based daily estimates of the Soil Moisture Index (SMI), generated by the JRC hydrological model LISFLOOD (https://github.com/ec-jrc/lisflood-code)^38^, also in use at the Copernicus European Flood Awareness System (EFAS, https://www.efas.eu). The LISFLOOD 6-hourly data are provided by the ECMWF as netCDF files, already referenced in EPSG:4326 geographic coordinates.

In Equation 1, ${I}_{T,Y}^{i}$ represents the SMI value at 18:00, namely:

2 $${I}_{T,Y}^{i}=\sum _{n\in T}\frac{SM{I}_{n,Y}^{i}}{N}$$

where SMI values range from 0 (extreme dry conditions) to 1 (extreme wet conditions) and $N$ is the number of days spanned by the time step **T**.

Champagne *et al*.^39^ suggest to use soil moisture baselines of 18–20 years or more to reliably estimate the relationship between high soil moisture and high crop yielding years. However, their results also indicate that even soil moisture data sets with a relatively short number of years of data can be used to identify soil moisture deficits that can negatively impact crop yields. In this work the soil moisture anomalies have been calculated using all the available data (1995 - present) as the reference baseline.

### Snow masks

The analysis of soil moisture variability across spatio-temporal scales is complicated by the presence of snow cover on the ground, which can make the model-based soil moisture anomalies unreliable. Indeed, the hydrological model currently in use has a simple implementation for snow processes based on a degree-day approach (https://ec-jrc.github.io/lisflood-model/). The reason is that the main purpose of LISFLOOD is to simulate high flows and floods and it is calibrated against discharge values. In this respect, future developments of the model are in progress, including the assimilation of snow cover data from satellite products. To overcome the above-mentioned limitations, the CDI integrates a snow cover masks dataset. This consists in a collection of gridded 10-day binary maps, where each pixel indicates the presence or absence of snow on the ground. The purpose of the snow cover masks dataset is excluding soil moisture anomalies from the computation of the CDI in pixels where snow is detected.

To generate the snow masks, we employed the NASA VNP10A1 product^40^, which provides daily snow cover tiles from 2012 to present. The snow data are derived from radiance observations acquired by the Visible Infrared Imaging Radiometer Suite (VIIRS) and are represented by values in the range of 0–100 of the Normalized Difference Snow Index (NDSI^41^). In this work, we have discarded NDSI values in the 1–10 range as uncertain, to reduce possible snow detection errors. This is the same rule employed by NASA for the generation of the 8-day snow maps MOD10A2 based on MODIS snow data retrieval (https://modis-snow-ice.gsfc.nasa.gov). The data have been downloaded from the HTTPS filesystem of the National Snow & Ice Data Center (NSIDC, https://nsidc.org). The VNP10A1 product consists of tiles of data gridded in the sinusoidal projection (375 m) and available as HDF-EOS5 archives. To re-project the files, first we used the command-line tool h5dump (https://www.hdfgroup.org/) to obtain the projection parameters and the upper-left, lower-right corner coordinates of each snow cover tile. Having this information, we used the R terra package^42^ to move each tile from the sinusoidal grid to the EPSG:4326 reference system.

To composite 10 days of VNP10A1 tile maps, we employed the following rules:

- a pixel is mapped as snow if snow has been found in that pixel for at least three days out of ten;
- a pixel covered by cloud (missing value) in a 10-day **T** composite is replaced (imputed) with its observed value at time step **T-1**;
- the resulting 10-day composites are resampled to the spatial resolution of the CDI (5 km) through a modal (most frequent observation) approach.

**Fig. 3:**
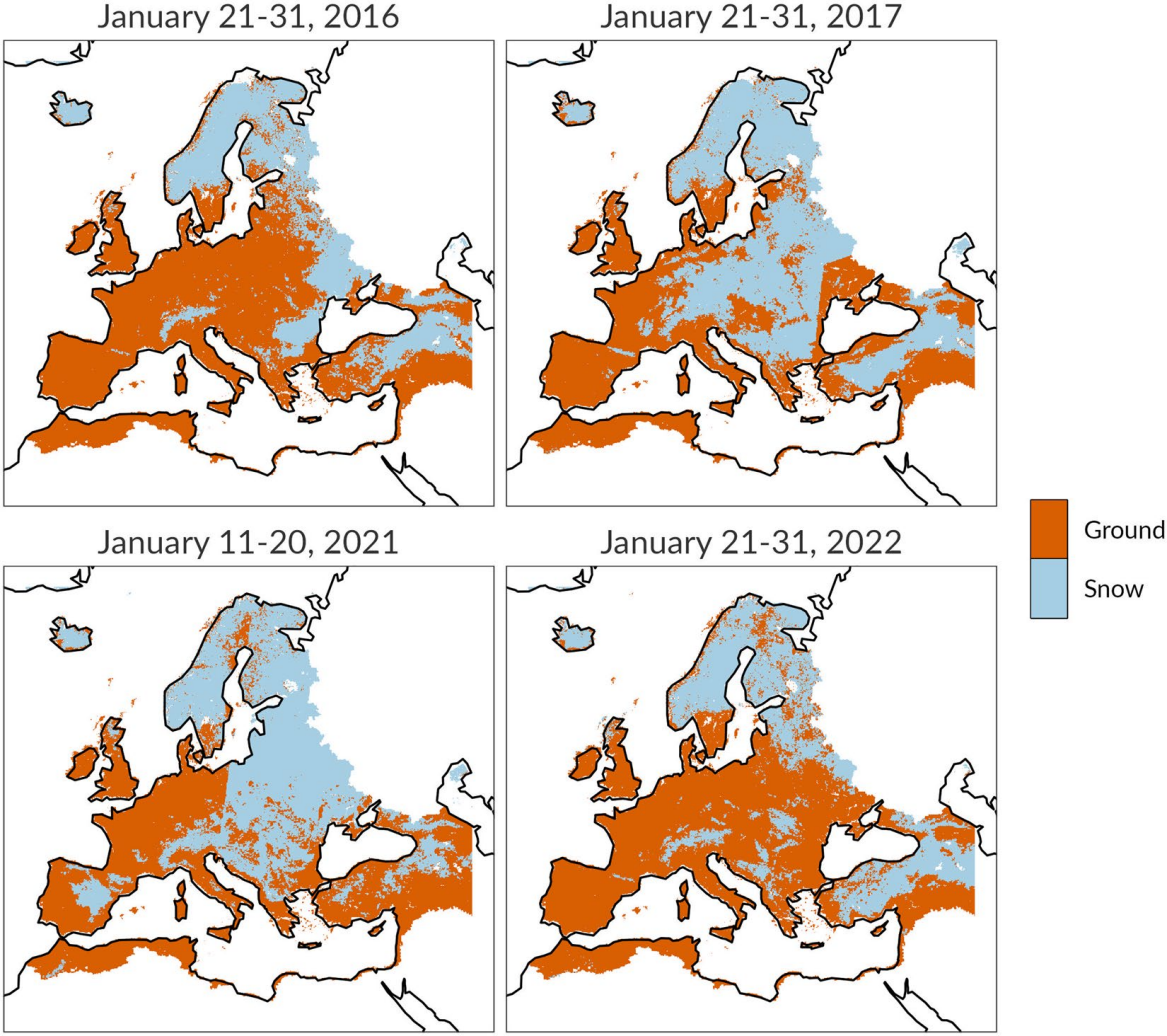
10-day periods with maximum snow cover extent in Europe in 2016, 2017, 2021 and 2022. Europe's 2015/2016 winter was characterized by the lack of snow for all of Europe (top-left map). January 2017 was a period of exceptionally cold and snowy weather in Eastern and Central Europe (top-right map). In January 2021 an unusual heavy snowfall in Spain was brought by the Storm Filomena (bottom-left map). In January 2022 the cold weather hit Athens and forced the closure of Istanbul airport (bottom-right map).

Figure 3 shows an example of the final 10-day snow masks for four different time steps and years.

The conceptual framework introduced in Table 3 outlines the calculation process of the CDI when all the three input anomaly indicators (SPI, SMA, FAPAR) are considered. However, the introduction of snow masks implies the exclusion of SMA where the pixels are masked (pixels with snow). Furthermore, if we also (reasonably) assume that in those pixels negative vegetation anomalies cannot occur, then the CDI conceptual framework boils down to the first two columns (A and B) of Table 3, where both negative SMA and FAPAR anomalies are not involved.

### Fraction of the absorbed photosyntetically active radiation anomalies

The Photosynthetically Active Radiation (PAR) is the incoming solar radiation in the spectral range from 400 to 700 nanometers^43^. Terrestrial vegetation needs this radiation for photosynthesis, namely to produce organic material from mineral components. FAPAR is the fraction of PAR (a ratio that ranges from 0 to 1 with no units) that is actually absorbed by vegetation and is commonly employed as an indicator of the state and photosynthetic activity of the vegetation^44^. However, as variations in vegetation state may be the result of several stress factors (not only precipitation and soil moisture deficits, but also plant diseases, pests, hail, flooding and so on), for drought monitoring it is crucial to interpret FAPAR anomalies along with the information provided by other drought-related indicators (indeed, as in the case with the CDI).

In this work, the FAPAR anomaly indicator is computed using the global VNP15A2H product from VIIRS^45^, freely available through the Land Processes Distributed Active Archive Center (LP DAAC, https://lpdaac.usgs.gov). This is an 8-day composite which provides FAPAR values on the sinusoidal projection at 500 meter spatial resolution. To reproject the data to the EPSG:4326 reference system, we followed the same procedure discussed for the VNP10A1 snow cover tiles.

To move from an 8-day to a 10-day composite (the temporal step of the CDI), first, we masked low-quality data with the use of quality flags which accompany the VIIRS FAPAR products; secondly, we computed each 10-day composite at time step **T** by a weighted-mean of its temporally closest 8-day composites (inverse distance in time weighting approach). Finally, the resulting 10-day composites were spatially resampled to the resolution of the CDI through a bilinear interpolation approach^46^.

### Crop masks

In this work, we used crop masks to: (1) identify crop areas in the study domain; (2) limit the analysis of crop-FAPAR anomalies to the growing season. The adoption of crop masks in the CDI computation is motivated by the fact that the standard FAPAR product has no concept of cropping seasons. In regions where agricultural production has strong seasonal patterns, the soil surface experiences shifts from vegetation to bare soil (after crop harvest) which significantly affect the spectral response of cropland areas. This means that, if not properly masked, anomalous signals of FAPAR from exposed bare soil in crop fields risk to be confused with drought stress on vegetation, resulting in wrong “Alert” assessments.

The CDI crop masks are based on the cropland and rangeland mask available through ASAP (Anomaly hot Spots of Agricultural Production, https://agricultural-production-hotspots.ec.europa.eu/), an early warning system that provides timely information about possible crop production anomalies^47^. The ASAP global cropland and rangeland is a raster file (spatial resolution of about $1{{\rm{km}}}^{2}$), where each pixel furnishes the percentage (values in the range 0–100) of crop/rangeland (area fraction). For the implementation of the crop masks in the CDI, first the ASAP cropland and rangeland mask were converted to a binary mask (“crop/no-crop” pixel) and the rangeland pixels filtered out. In the resulting binary mask, a pixel was mapped as crop if it contains at least 1% of crop. Then, the binary mask was integrated with the information retrieved by the ASAP phenology maps. For each crop cell, these maps indicate the Start Of the growing Season (SOS) and the End Of the growing Season (EOS). These two pieces of information are reported at a temporal scale of 10 days. Merging the binary crop mask with the information about the SOS and EOS, we generated a collection of 36 binary maps (three per month, as already described in Table 4) where: 0 identifies a cropland out of the growing season; 1 a cropland in the growing season.

Note, while the snow masks are dynamic maps, the CDI crop masks are “static”, given that the start and the end of the growing season of each crop cell are fixed in time (don’t change from one year to the other).

Similarly to what explained for snow masks, the use of crop masks implies that negative FAPAR anomalies are excluded from the computation of the CDI when crops are out of the growing season. The corresponding conceptual framework is given by the columns A, B, C and F in Table 3, where negative FAPAR anomalies are not involved.

## Data Records

The dataset is available through the Copernicus European Drought Observatory web site (https://drought.emergency.copernicus.eu)^48^ as GeoTIFF or netCDF files, under a Creative Commons Attribution 4.0 License. The netCDF file format is intended to efficiently store multiple temporal layers in one file. For users who have not experience with netCDF archives, the CDI maps are also available as single GeoTIFF files. The CDI raster files have a spatial resolution of 5 km, a temporal resolution of 10 days and are available from 2012 to present. The coordinate reference system is EPSG:4326. All data files are accompanied by metadata, which provide a description of what the data represent, their spatial and temporal coordinates. In this respect, our netCDF products follow the Climate and Forecast Metadata Conventions^49^.

Following a common convention for signaling danger levels^50^, in the CDI colour palette (see Table 2) the increasing drought severity from Watch to Alert is represented through warm colors (yellow, orange and red).

Through the scientific background pages of the European Drought Observatory and Global Drought Observatory, final users can access and download the indicator factsheets (https://drought.emergency.copernicus.eu/data/factsheet) which contain the most relevant information about the computation of the CDI and its input indicators (SPI, SMA and FAPAR).

## Technical Validation

The responsibility of maintaining the quality of input data (precipitation, soil moisture and vegetation) lies with the data providers. Conversely, the quality of the anomaly indicators and of each final CDI map is screened for errors by the scientific team behind the European Drought Observatory. Suspect values in the data records are investigated with the help of R and Python scripts, while visual inconsistencies in the generated maps are discussed among all members of the team before their publication. As for the the capability of the CDI to accurately reproduce the evolution of previous drought events, Cammalleri *et al*.^23^ have analysed three major drought episodes in central Europe (2003), the Iberian Peninsula (2005), and northern Europe (2018). Their analysis showed that the CDI framework provides a realistic description of the spatiotemporal characteristics of such events, with a realistic succession of the Watch, Warning, and Alert stages and a large spatial consistency in the modelled spatial patterns.

We use this section to show the correct implementation of the snow masks and crop masks in the CDI computation. More specifically, we illustrate that the application of the crop and snow masks diminishes unreliable spatial patterns in the CDI maps, leading to a general improvement of the agricultural drought assessment without fundamentally altering the whole behaviour of the CDI over time. We carry out the validation exercise through the visual inspection of the CDIv4 data compared to previously published maps (CDIv2 and CDIv3). To conclude this section, we present evidence that the assessment of drought events according to the CDIv4 is in line with other specialized drought indicators.

**Fig. 4:**
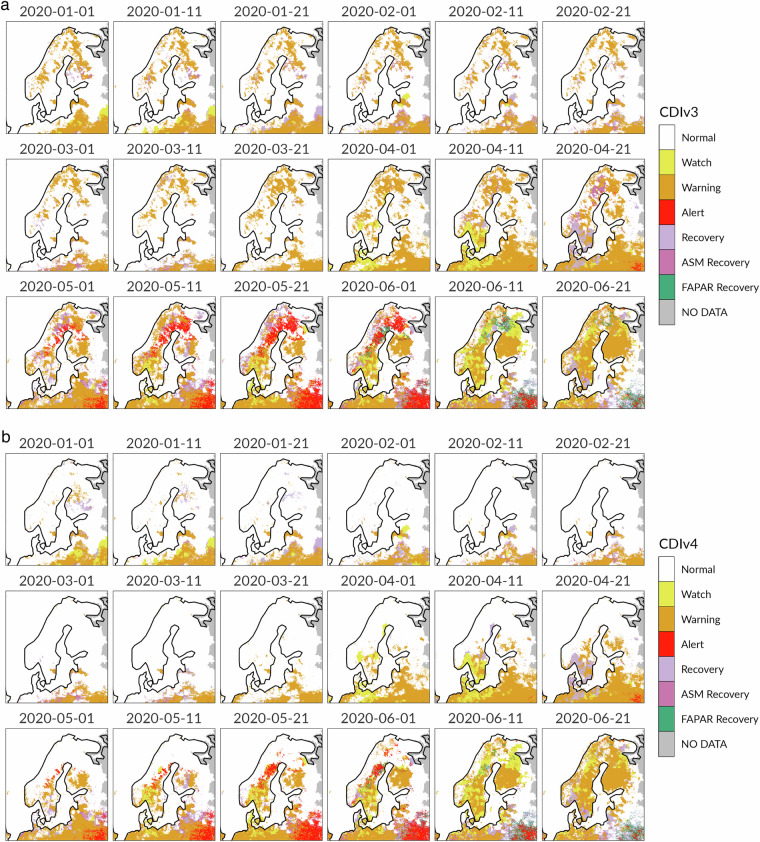
Combined Drought Indicator version 3 (upper panel) and version 4 (lower panel) for Finland, Sweden and Norway during the 1st to the 18th 10-day period in 2020 (January - June).

**Fig. 5:**
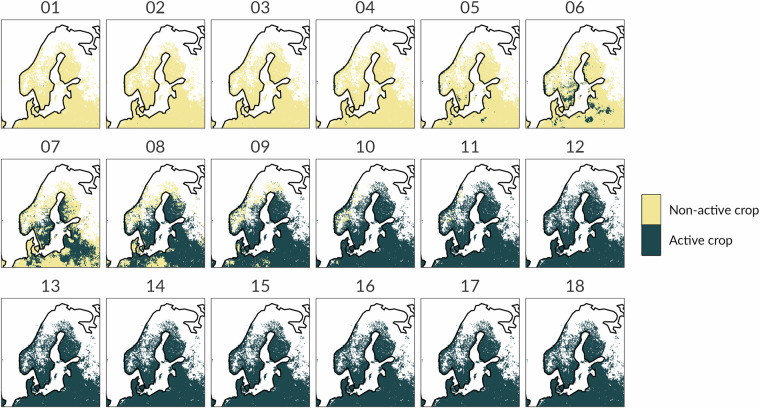
Static crop masks for Finland, Sweden and Norway during the 1st to the 18th 10-day period in a year (January - June).

The first example concerns with the spatio-temporal evolution of the CDI in three Nordic countries (Finland, Sweden and Norway), located in the most northern growing region of field cultivation in the world and characterized by a relatively small crop production^47,51^. The period of interest is the first six months of year 2020, when the CDIv3 (Fig. 4a) exhibits severe dry conditions, with several pixels assigned to the “Warning” class (soil moisture deficit) gradually evolving to the “Alert” class (vegetation stress). Finland, Sweden and Norway are characterized by one growing season which typically starts in late March/early April and terminates in November (Figure not shown). The eighteen 10-day maps in Fig. 5 display the spatial distribution of the crop fields (active and non-active crop fields) in the January-June period. A visual analysis of these maps highlights that most of the pixels in Alert condition since early May 2020 are associated to negative FAPAR anomalies in active crop fields. However, part of these pixels (especially in the northern part of Sweden and Finland) also depend on negative FAPAR anomalies from vegetation other than crop.

**Fig. 6:**
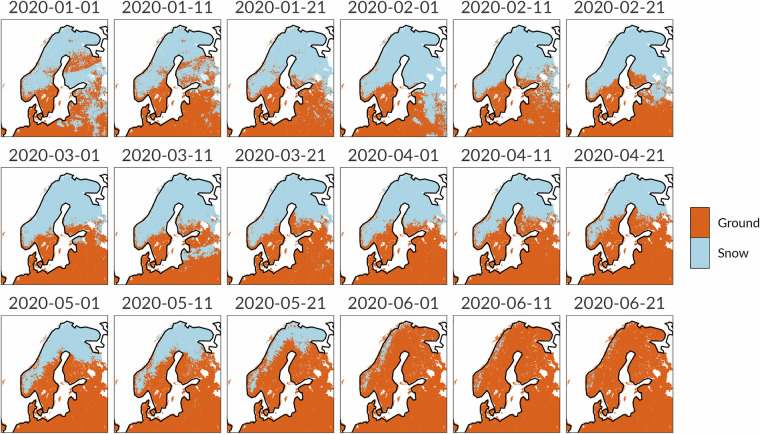
Dynamic snow masks for Finland, Sweden and Norway during the 1st to the 18th 10-day period in 2020 (January - June).

As explained in the Snow Masks subsection, the soil moisture anomalies based on the hydrological model LISFLOOD may have some issues in snow covered areas (Fig. 6). The result is that the drought signals in Fig. 4a can convey misleading messages to final users. The CDIv4 overcomes this issue through the incorporation of the snow masks, which simplify the CDI conceptual framework from Table 3 to a subtable of columns A and B. The maps in Fig. 4b demonstrate that, where the snow masks are applied, the Warning and Alert signals disappear as expected. However, when negative soil moisture anomalies appear without snow ground (e.g. in June 2020), the spatial patterns of the CDIv3 and CDIv4 maps are almost identical.

**Fig. 7:**
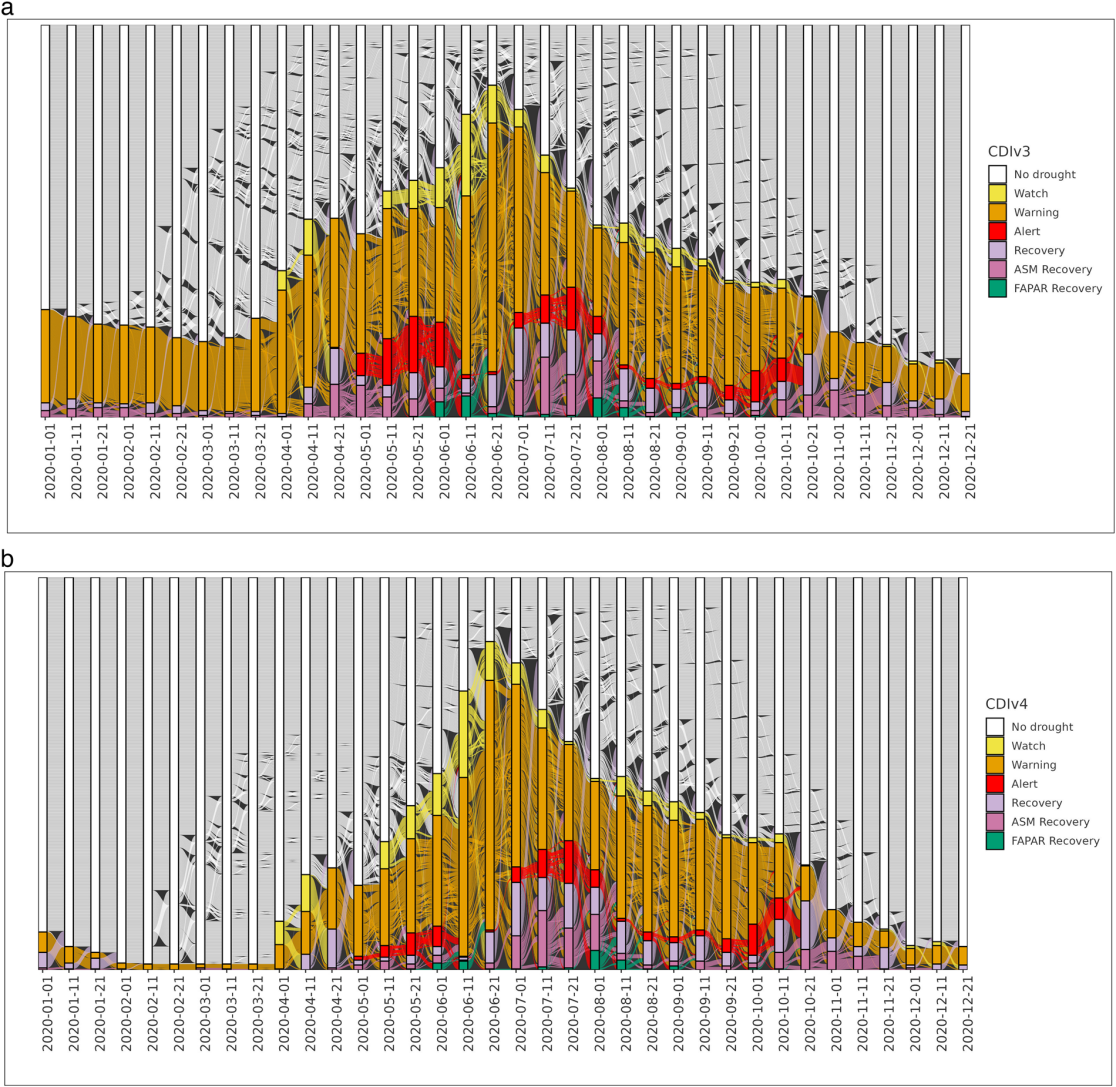
Alluvial plot of the Combined Drought Indicator for Finland, Sweden and Norway during the 1st to the 36th 10-dayperiod in 2020 (January - December).

The alluvial plots in Fig. 7 depict the dynamic behaviour of the CDIv3 (9) and CDIv4 (10) for Finland, Norway and Sweden throughout the year 2020. In both diagrams, the vertical blocks represent the 10-day time steps **T** when the CDI maps are computed. The lines between two adjacent blocks visualize the evolution (flow) of each pixel in a CDI map, transitioning from one drought category (No drought, Watch, Warning, Alert, etc.) to another over time. These transitions reflect changes in anomalies of precipitation, soil moisture, and vegetation health status. Although an alluvial plot can look intimidating to an untrained eye, it allows us to condense a multitude of geospatial data points (specifically, the pixels in the 36 CDI maps of 2020) into a single comprehensive plot that effectively highlights the dynamic nature of the CDI over time. In addition, it allows for a rapid visual assessment of whether the CDI conceptual framework is correctly implemented, highlighting transitions that are not allowed in the conceptualization. By comparing the two alluvial plots, we see that the temporal evolution of the CDIv3 and CDIv4 data across 2020 is almost identical. At the same, it is apparent the effect of the snow masks in the CDIv4 alluvial plot, resulting in a lower number of Warning occurrences. We conclude the analysis of this first example by noting that the visual inspection of the alluvial plots confirms that the pixels transition from one state to another according to the conceptual frameworks outlined in Table 3.

**Fig. 8:**
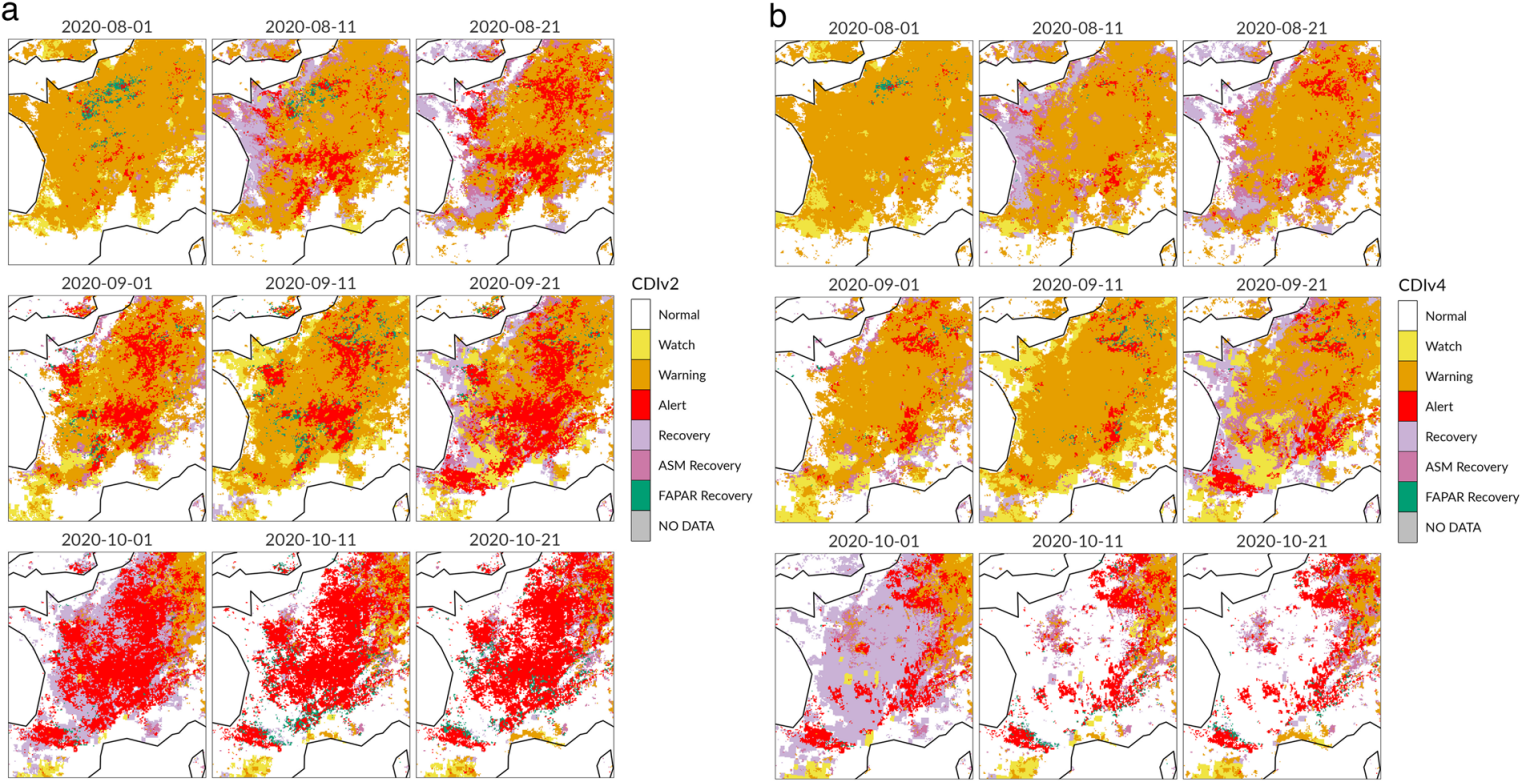
Combined Drought Indicator for France during the 22nd to the 30th 10-day period in 2020 (August - October).

**Fig. 9:**
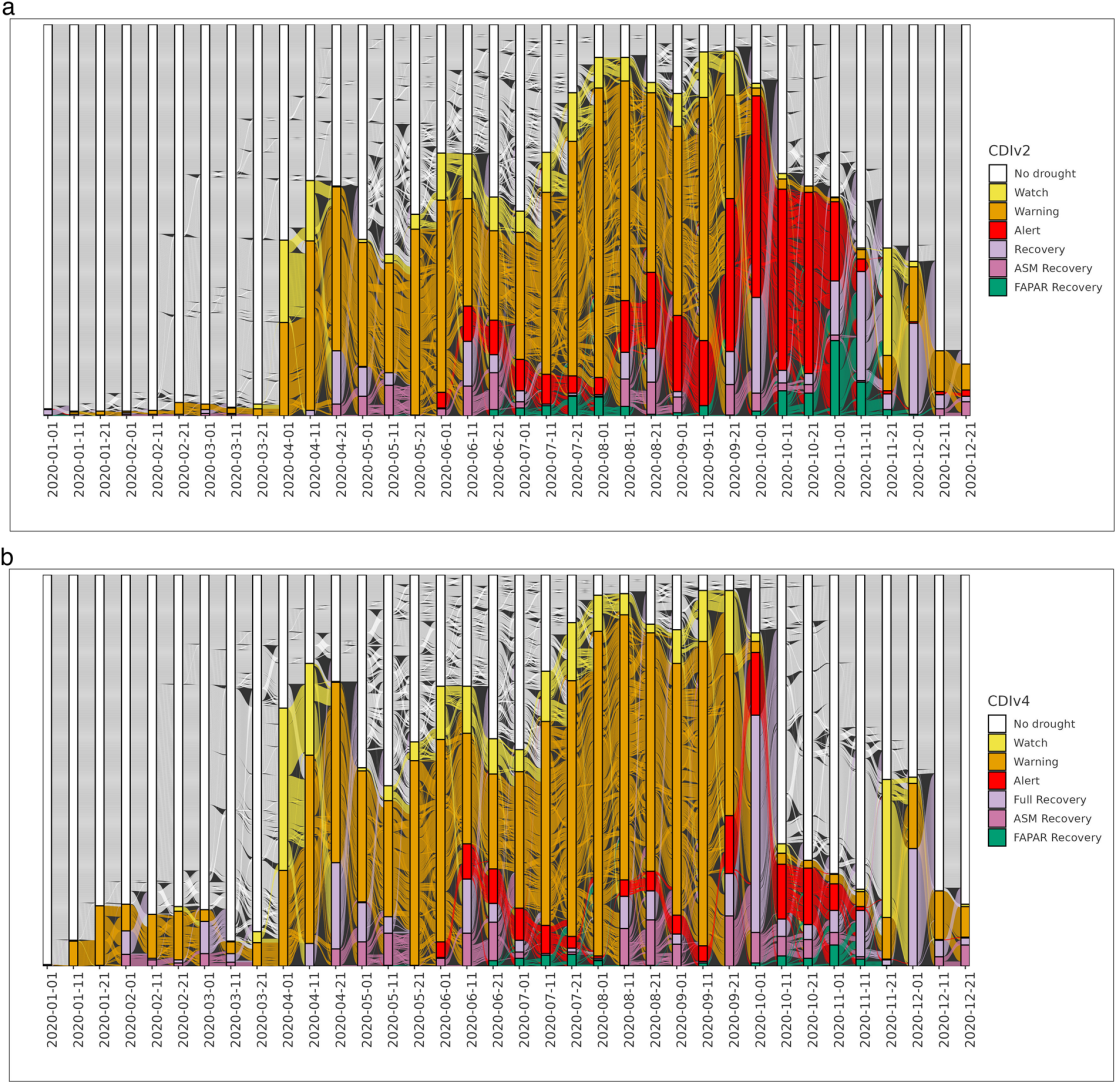
Alluvial plot of the Combined Drought Indicator for France during the 1st to the 36th 10-day period in 2020 (January - December).

The second example focuses on the use of the crop masks to filter out negative vegetation anomalies in France from August to October 2020. When the CDI does not integrate the crop masks (CDIv2), a significant portion of the country is affected by below-normal FAPAR anomalies (Fig. 8a). At the same time, the CDI crop masks (Figure not shown) indicate that most of the crop fields across France in the period August-October are out of the growing season, and the corresponding negative FAPAR anomalies should not be attributed to crop stress. The incorporation of the crop masks in the CDI computation (Table 3, columns A, B, C and F) results in a general decrease in the Alert occurrences (Fig. 8b). The most notable change involves the maps for October 2020. In the initial 10-day period of October, the Alert signals in the CDIv2 maps correspond to Recovery signals in the CDIv4 maps. More interestingly, the Alert signals visible across the central part of France during the second and the third 10-day periods of October 2020 correspond to “No Drought” conditions in the CDIv4 maps. This result is clearly visible in the right side of the alluvial plots in Fig. 9.

**Fig. 10:**
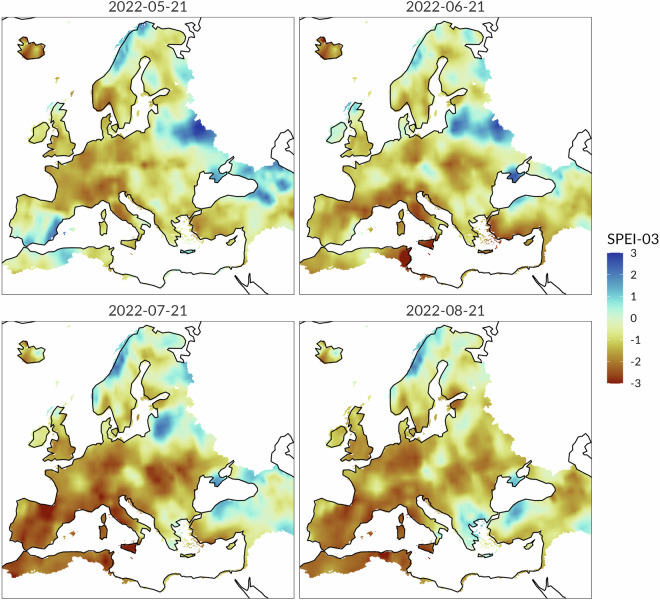
Standardized Precipitation Evapotranspiration Index (SPEI-3) from May to August 2022.

**Fig. 11:**
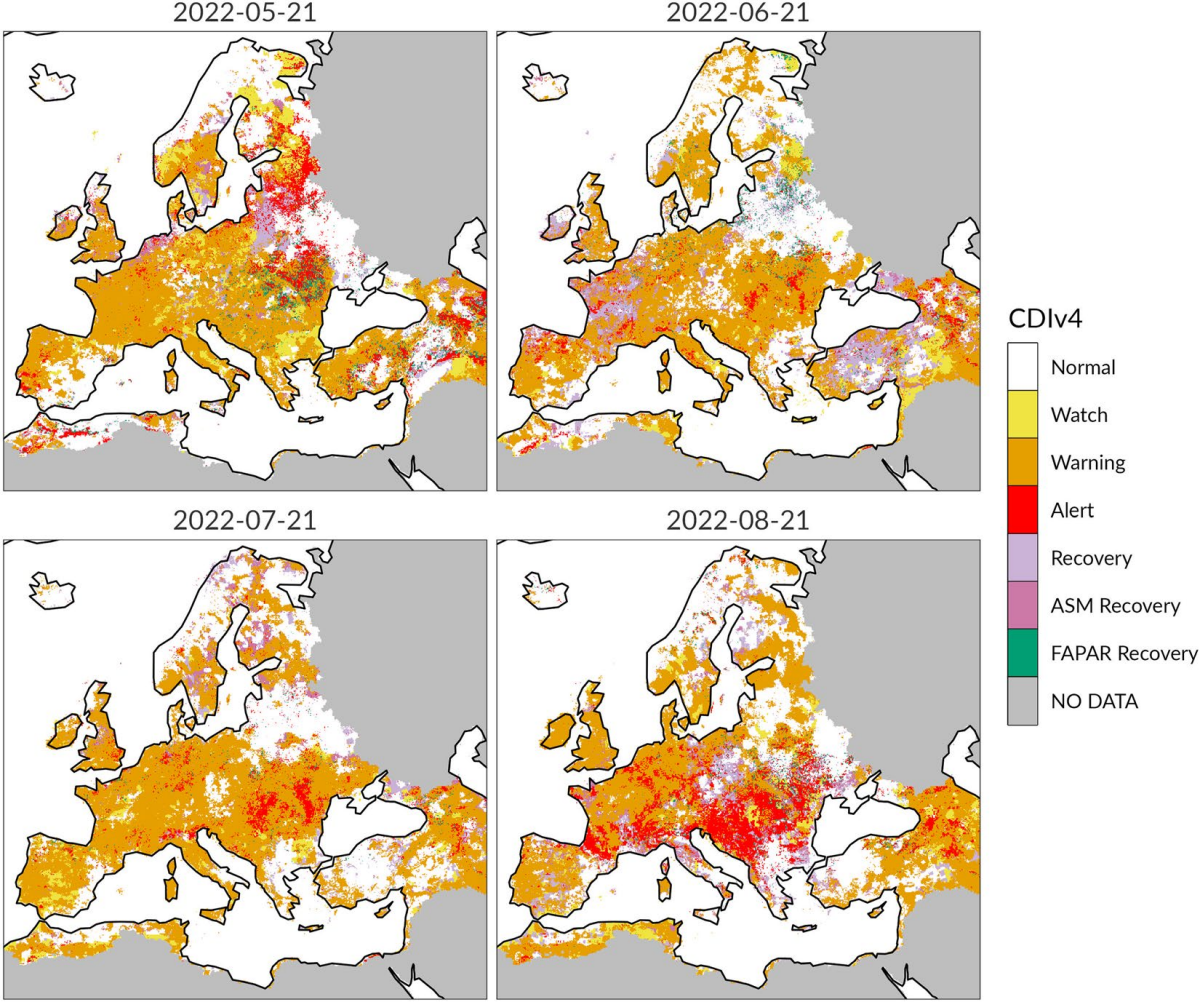
Combined Drought Indicator (CDIv4) from May to August 2022.

The following example shows that the CDI allows to draw conclusions about drought severity and spatial extent that are consistent with those derived from other specialized indicators. Specifically, the Figs. 10 and 11 compare the Standardized Precipitation Evapotranspiration Index (SPEI^52^) and CDI maps from May to August 2022. The SPEI is an indicator that is conceptually similar to the SPI, but which incorporates both precipitation and potential evapotranspiration (PET) data into its computation. In situations where specific information on soil moisture conditions is not available, the SPEI can be used for identifying soil moisture droughts^53^. Interestingly, this characteristic makes the SPEI to be somewhat comparable to the CDI. In the proposed example, the monthly 3-month Standardized Precipitation Evapotranspiration Index (SPEI-3) data have been obtained from the Global SPEI database (https://spei.csic.es/), which offers long-time, robust information about drought conditions at the global scale.

The visual analysis of the maps in Figs. 10 and 11 indicates that the spatial patterns of the SPEI-3 and the CDI exhibit a high degree of similarity. Specifically, the spatial distribution of the negative anomalies in the SPEI-3 maps closely mirrors the spatial distribution of the three primary drought CDI classes (Watch, Warning and Alert). Conversely, areas characterized by recovery/no drought conditions according to the CDI correspond well with positive anomalies in the SPEI-3 maps. For example, very good agreement between the two indicators is visible over the Mediterranean and the Baltic regions, while main differences are apparent in Iceland. The proposed example suggests that the SPEI-3 allows to properly distinguish and delineate a drought event in terms of a simplified water balance and also provides crucial information about drought severity. However, the CDI maps provide more detailed information about the evolution and the stage of drought. The reason is that the CDI uses an effective classification scheme based on drought and recovery classes. The inclusion of recovery classes is particularly valuable for monitoring and communication, addressing an aspect of drought that is often overlooked or neglected by drought indicators. The CDI classification scheme based on both drought conditions and recovery classes is a relevant feature of the CDI, which can support policy-makers in effective risk management and decision-making. The CDI conceptual framework allows to readily interpret the stages of drought and their evolution over time based on the CDI thresholds.

## Data Availability

The Python code for computing the Combined Drought Indicator is available in the DROUGHT-CDI repository at https://code.europa.eu/drought/edo_gdo/drought-cdi. This repository also contains an example of input data for generating the CDI maps for the year 2018. For detailed instructions and dependencies, please refer to the README file in the repository.
